# The Use of Glucagon-Like Peptide-1 (GLP-1) Agonists in the Perioperative Period: A Case Study

**DOI:** 10.7759/cureus.77106

**Published:** 2025-01-07

**Authors:** Todd R Anderson, Mark Bushhouse, Emily J Carletto, Kelly Holder

**Affiliations:** 1 Medicine, Campbell University School of Osteopathic Medicine, Lillington, USA; 2 Obstetrics and Gynecology, Campbell University School of Osteopathic Medicine, Buies Creek, USA; 3 Obstetrics and Gynecology, Campbell University School of Osteopathic Medicine, Lillington, USA

**Keywords:** benign gyn, diabete mellitus, glp-1 receptor agonists, ob-gyn, post-op complications, postoperative ileus, small bowel surgery

## Abstract

Glucagon-like peptide-1 (GLP-1) receptor agonists, initially developed for type 2 diabetes mellitus (T2DM), have demonstrated efficacy in improving glycemic control and promoting weight loss. However, their use is associated with gastrointestinal side effects, including delayed gastric emptying, which can complicate perioperative care. Current guidelines recommend discontinuing GLP-1 agonists before surgery to reduce risks such as regurgitation and aspiration. Despite these preoperative recommendations, guidance on reintroducing these medications postoperatively is sparse.

This case study discusses a 55-year-old woman with diabetes and uterine fibroids who underwent an elective total laparoscopic hysterectomy with bilateral salpingectomy. Following surgery, the patient resumed a GLP-1 agonist on postoperative day one without specific instructions. Ten days later, she presented with symptoms of nausea, vomiting, and abdominal pain, raising concerns for postoperative ileus or small bowel obstruction (SBO). Imaging revealed delayed transit of enteric contrast, initially interpreted as SBO, leading to a scheduled exploratory laparotomy. However, subsequent radiographic findings and clinical improvement led to the cancellation of surgery, with the patient recovering under conservative management.

This case highlights the diagnostic challenges in distinguishing between postoperative ileus, SBO, and GLP-1-induced delayed gastric motility. The overlap in symptoms and imaging findings underscores the importance of cautious clinical assessment to avoid unnecessary surgical interventions. Additionally, it raises critical questions about the appropriate timing, dosing, and monitoring of GLP-1 agonists in the postoperative period.

The case emphasizes the need for robust guidelines on postoperative GLP-1 agonist management. With the growing use of these medications, further research is necessary to determine optimal strategies for their reintroduction after surgery, balancing the benefits of metabolic control with the risks of gastrointestinal complications.

## Introduction

Glucagon-like peptide-1 (GLP-1) receptor agonists are medications originally developed for managing type 2 diabetes mellitus (T2DM). They mimic the action of the endogenous incretin hormone GLP-1, which plays a critical role in glucose homeostasis. By stimulating GLP-1 receptors, these agents enhance glucose-dependent insulin secretion, suppress glucagon release, slow gastric emptying, and promote satiety. This multifaceted mechanism improves glycemic control and supports weight reduction, making GLP-1 agonists increasingly valuable in managing obesity and metabolic syndrome [1,2]. Given their growing indications, GLP-1 receptor agonists are now used by a broader patient population, including patients with obesity without diabetes mellitus.

Despite their therapeutic benefits, GLP-1 and GIP receptor agonists are not without adverse effects. The most common side effects include gastrointestinal symptoms such as nausea, vomiting, and delayed gastric emptying, leading to constipation. Rare but serious adverse events include pancreatitis, gallbladder disease, and potential thyroid C-cell tumors. Patient selection and monitoring are crucial to minimize risks and optimize outcomes [3,4].

The American Society of Anesthesiologists has recommended holding GLP-1 and GIP agonists a week before elective surgeries to reduce the potential risk of regurgitation and pulmonary aspiration during the procedure [5-7].

A common complication of surgery is postoperative ileus, which can range anywhere from two to seven days [8]. The incidence of postoperative ileus after hysterectomies for benign indications is around 9% [9]. Another common etiology for delayed gastrointestinal motility or the inability to pass flatus or bowel movement after surgery is small bowel obstruction caused by peritoneal adhesions that can entrap portions of the bowel. Small bowel obstructions are a common complication of abdominal surgery, which is estimated to occur in around 9% of abdominal surgery cases [10]. A small bowel follow-through study is used for the evaluation of both of these conditions in the postoperative period. This study involves the patient ingesting barium contrast material to visualize the route of the gastrointestinal path via serial radiographs [11]. The time it takes for the contrast to travel through the gastrointestinal tract is anywhere from one to four hours [11,12]. Prolonged transit time raises concerns for possible obstructive or severe ileus. The treatment for postoperative ileus is typically conservative and includes intravenous fluids, electrolyte replacement, non-opioid pain management, early ambulation, and slow resumption of an oral diet [8,13]. On the other hand, the treatment for small bowel obstruction is bowel rest and gastric decompression, and if conservative measures fail, a bowel resection surgery might be indicated [10,14].

This report examines a challenging case of postoperative ileus in the setting of GLP-1 and GIP agonist use during the early postoperative period.

## Case presentation

This case presents a 55-year-old woman who presented to the emergency department with intermittent abdominal pain and vaginal bleeding for the last three days. The patient had a past medical history of uterine fibroids, menometrorrhagia, anemia, asthma, and diabetes mellitus with three previous cesarean deliveries. The patient reported taking canagliflozin and tirzepatide for the past four months to aid in the treatment of her recent diabetes mellitus diagnosis. Her recent dose of tirzepatide was 10 mg weekly. The patient was evaluated in the emergency department and discharged home with appropriate pain medications and a follow-up appointment with her gynecologist.

At the patient's follow-up appointment, she underwent an endometrial biopsy for evaluation of the abnormal uterine bleeding. The tissue pathology confirmed benign atrophic inactive endometrium with no identified hyperplasia, malignancy, or endometritis. The patient underwent a pelvic sonogram identifying a uterus measuring 4.9 cm 4.3 cm x 7.5 cm with multiple fibroids, with the largest fibroid measuring 2.3 cm x 2.3 cm x 2.3 cm. The patient was offered a total laparoscopic hysterectomy with bilateral salpingectomy, and, with shared medical decision-making, the patient agreed to undergo the procedure. During her pre-operative appointment, the patient was advised to stop taking tirzepatide and to schedule elective surgery two weeks from the last dose of tirzepatide.

Two weeks later, the patient presented to the hospital for her elective total laparoscopic hysterectomy with bilateral salpingectomy. During the surgery, omental adhesion to the anterior abdominal wall and dense adhesion between the lower uterine segment and bladder was noted, and an adhesion lysis procedure was performed before the hysterectomy portion of the case. Otherwise, normal-appearing tubes, ovaries, and uterus were noted. The surgery was completed without complications, and the patient tolerated the procedure well.

The patient was transferred to the PACU in a stable condition, and on postoperative day one, she had stable vital signs, appropriate pain control, and minimal vaginal bleeding. The patient was discharged home with postoperative return precautions, including fevers, chills, severe abdominal pain, and heavy bleeding. She was not provided specific instructions regarding the timing or the dose of her GLP-1 and GIP agonist reinitiation. When the patient got home, she restarted her weekly injection of tirzepatide 10 milligrams.

On postoperative day 10, the patient presented to the emergency room with reports of nausea, vomiting, and diffuse abdominal pain. STAT CT scan showed borderline distended small bowel loops with a possible transition point in the right pelvis. The patient reported that she initially felt well after being discharged but then began experiencing nausea, vomiting, and diffuse abdominal pain. The patient endorsed less frequent bowel movements and hard, small-caliper stools. The patient denied passing flatus since her operation. 

**Figure 1:**
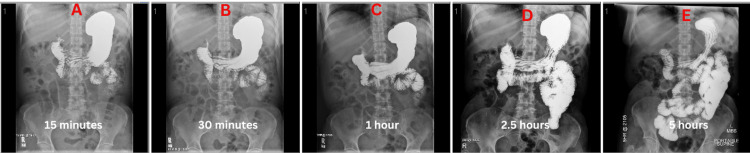
Small bowel follow through demonstrating delayed motility of barium contrast with the respective time stamps A) 15 minutes after the start of the small bowel series; B) 30 minutes after the start of the small bowel series; C) 1 hour after the start of the small bowel series; D) 2.5 hours after the start of the small bowel series; E) 5 hours after the start of the small bowel series.

**Figure 2:**
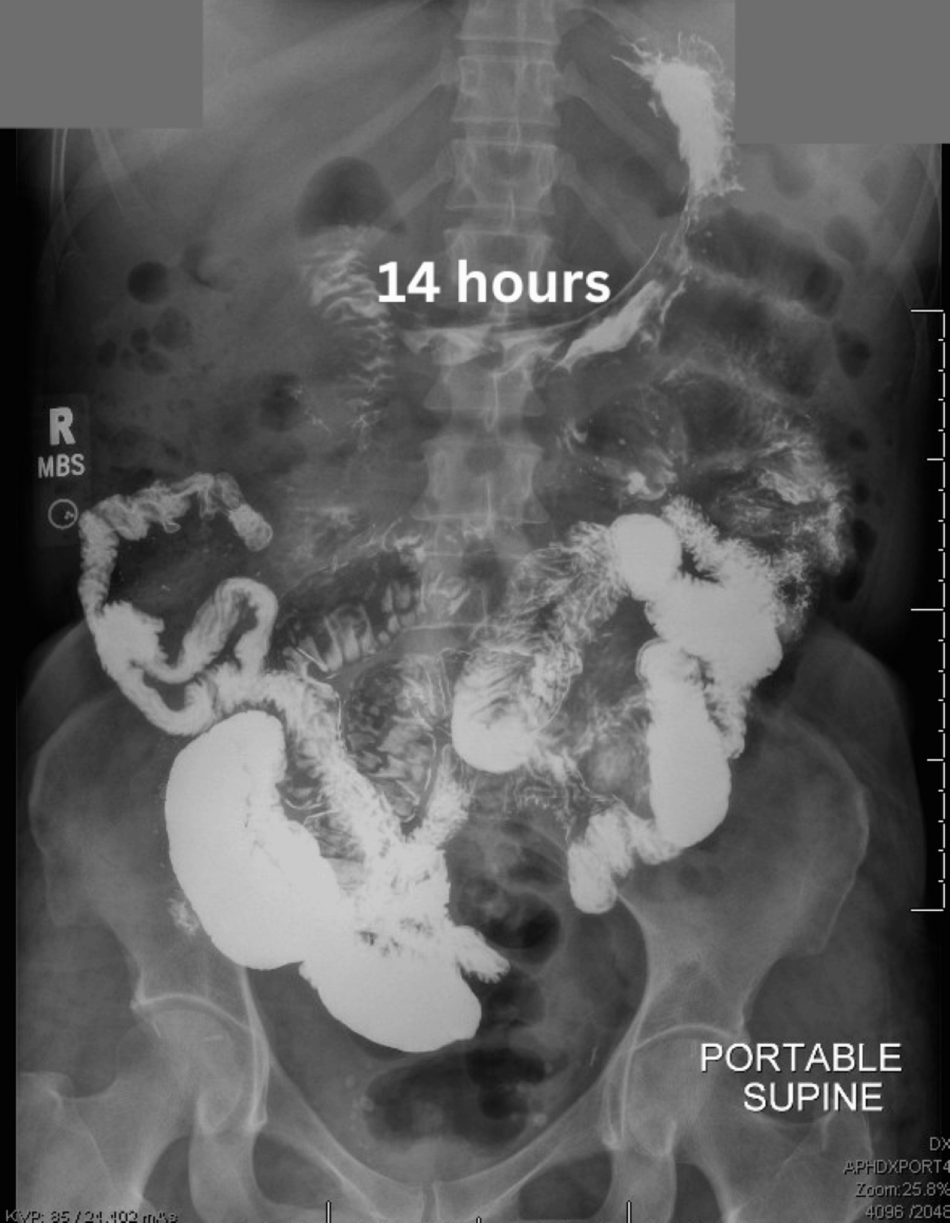
Abdominal radiograph demonstarting barium contrast within the distant small intestines and proximal large intestine at 14 hours

The patient was admitted to the hospital under the working diagnosis of postoperative ileus. The differential diagnosis included postoperative ileus vs. small bowel obstruction vs adverse effects of tirzepatide. General surgery was consulted, and recommended trending electrolytes, keeping the patient nothing per mouth (NPO), and obtaining a small bowel series (Image 1) and an X-ray of the abdomen and pelvis. The small bowel series revealed a slow passage of enteric contrast through the small bowel. Imaging from the small bowel series was taken at 15 minutes, 30 minutes, 1 hour, 2.5 hours, and 5 hours. Five hours following ingestion of the enteric contrast, it had yet to reach the colon (Figure 1E). The X-ray of the abdomen and pelvis demonstrated contrast medium in the stomach and small bowel; however, no contrast was observed within the colon, and several loops of the small bowel were dilated (Figure 2). Imaging studies raised suspicion for a small bowel obstruction, but clinical findings were equivocal. 

On the second day of admission, postoperative day 11, the patient reported having better pain control; however, she still endorsed pain in her right lower abdominal quadrant and right lower back. The patient reported being able to pass gas but had not been able to have a bowel movement since admission. General surgery reviewed the films and believed that there was a small bowel obstruction and scheduled an exploratory laparotomy with a possible small bowel resection. 

**Figure 3:**
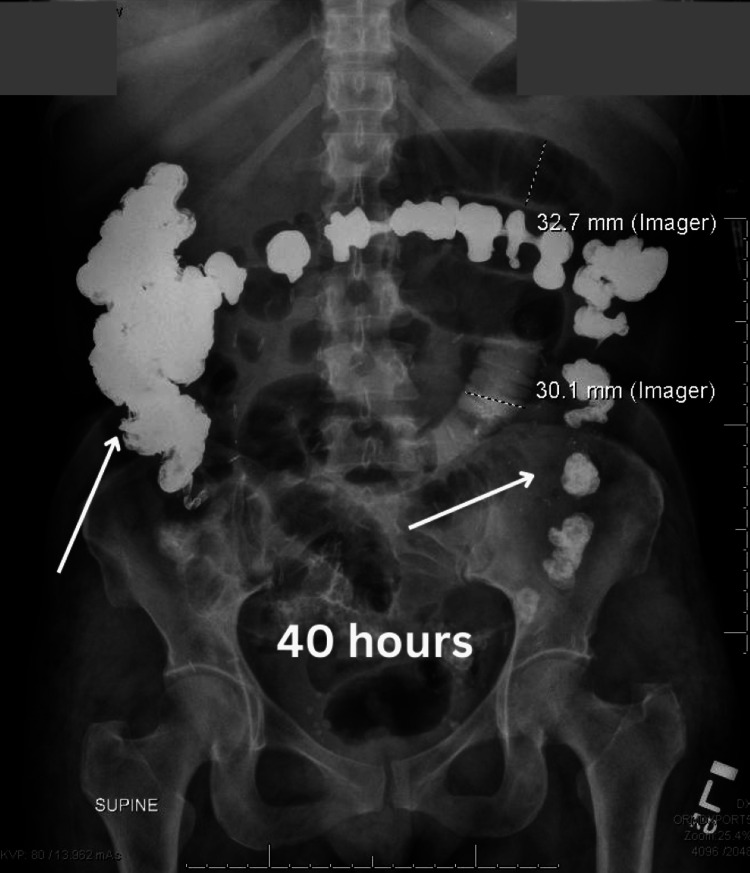
Abdominal radiograph demonstrating barium contrast located in the colon at 40 hours after small bowel follow through

On the day of the surgery, postoperative day 12, the general surgery team ordered a repeat abdominal radiograph that demonstrated contrast in the colon, with the majority of the contrast within the hepatic flexure, 40 hours after the initiation of the small bowel series (Figure 3).

**Figure 4:**
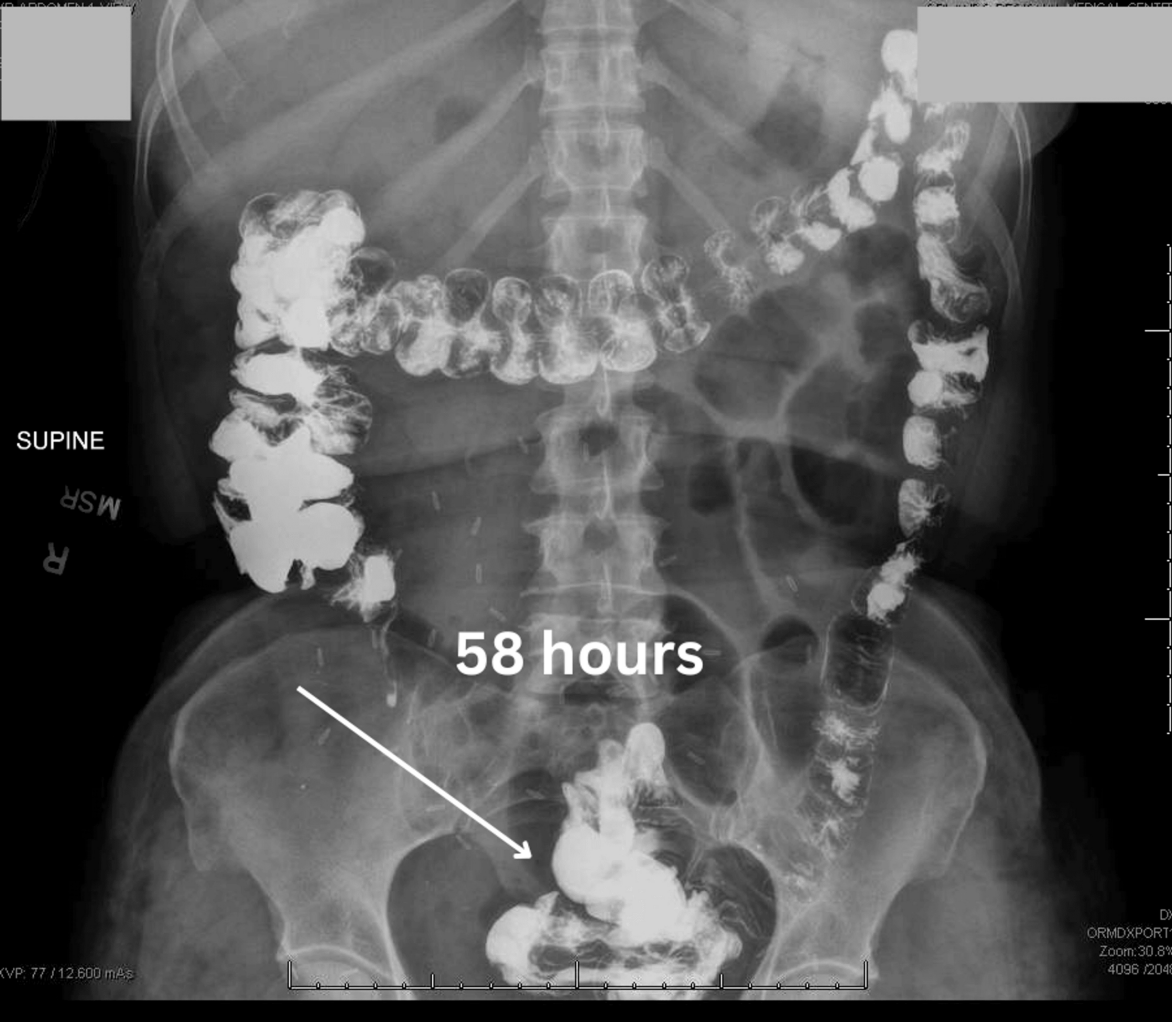
Abdominal radiograph demonstrating barium contrast in the distal colon at 58 hours after the start of the small bowel follow-through

That morning, the patient reported passing flatus. The exploratory laparotomy was canceled, and the patient continued conservative management. The next day, postoperative day 13, a repeat abdominal radiograph showed oral contrast within the distal colon and rectum and decreased gaseous distention of the small bowel 58 hours after the start of the small bowel series. (Figure 4) The patient's clinical status had improved, and the patient was discharged.

## Discussion

This case exemplifies the importance of the management of GLP-1 and GIP receptor agonists in the perioperative period. GLP-1 and GIP receptor agonists, being rather newer medications on the drug market, have not given the medical establishment enough time to establish guidelines for use, especially in the perioperative period. GLP-1 and GIP agonist dosing regimens are titrated to the most effective dose, usually making small increases over multiple weeks. The typical starting dose of tirzepatide is 2.5 milligrams per week for the first four weeks, then doubling the dose every four weeks until reaching a maintenance dose of 12.5-15 milligrams [15]. This patient had reported using this medication for the past four months and was currently on 10 milligrams per week dosing. The patient was counseled at her pre-operative appointment to stop taking the GLP-1 and GIP agonists two weeks before surgery, which is agreeable to the recommendation set forth by The American Society of Anesthesiologists [5]. 

The importance of this case comes during the postoperative period. A laparoscopic hysterectomy at most healthcare facilities in the United States is considered an outpatient procedure that does not require an overnight hospital stay. The postoperative milestones that a patient must meet to be able to be discharged home safely differ from provider to provider; however, a common set of postoperative milestones includes pain control and the ability to urinate freely. In this case study, the patient had good pain control and was able to void without issues, and was safely discharged by her provider. The patient was never advised when or how to restart her GLP-1 and GIP receptor agonists. The day after her surgery, postoperative day one, the patient restarted her GLP-1 and GIP receptor agonists at the dose she was previously on, 10 milligrams. The following week the patient reports nausea, vomiting, and constipation, which are well-documented adverse effects of this medication. The challenge for healthcare providers in the case was to distinguish between postoperative ileus, small bowel obstruction, or the adverse effects of GLP-1 and GIP agonists.

In the workup for postoperative ileus, which is a small bowel series, the transit time for the barium contrast from the oral cavity to the distal rectum is typically up to four hours [11,12]. In the case of this patient, the transit time from the oral cavity to the distal rectum was roughly 48 hours which was demonstrated over the small bowel follow-through and the two repeat kidney, ureter, and bladder (KUB) radiographs. The small bowel follow-through showed that the contrast had not reached the colon in eight hours, which was interpreted as a possible bowel obstruction or ileus. The consulted general surgery team reviewed the recent films and suggested an exploratory laparotomy for possible resection of the obstructed bowel. A repeat KUB was ordered to confirm the working diagnosis of small bowel obstruction on the morning of the scheduled surgery. However, this film demonstrated that there was contrast within the distal rectum. In addition, to the reassuring radiograph, the patient’s clinical condition improved, and the patient reported being able to pass stool.

However, in this case, the authors may not be able to prove that this postoperative complication was likely due to the use of GLP-1 and GIP receptor agonists in the early postoperative period. There is evidence to reject the diagnosis of small bowel obstruction due to the delayed transit of the contrast for the small bowel follow-through. In addition, in the possibility of postoperative ileus, the patient reported being able to stool, although hard and small in quality, in the early postoperative period, which would not be in line with the diagnosis of postoperative ileus.

Further research and guidance are needed for this new type of medication. Guidelines are available for the pre-operative period, but guidance on restarting the medication once the patient has been off it for multiple weeks is sparse. This case calls into question whether a patient restarts the medication after surgery and at what dose is safe. Another question that needs to be answered is at what point in the postoperative period.

## Conclusions

This case highlights the complexities of perioperative management of GLP-1 and GIP receptor agonists and the urgent need for robust guidelines, especially for their postoperative reintroduction. It underscores the diagnostic challenges in differentiating complications such as postoperative ileus, small bowel obstruction, and GLP-1 and GIP agonists effects, given overlapping symptoms like nausea, vomiting, and abdominal distention. Despite adherence to preoperative recommendations, the lack of clear postoperative guidance nearly led to unnecessary surgery, emphasizing the importance of conservative management and ongoing reassessment. With the expanding use of GLP-1 and GIP receptor agonists for diabetes mellitus and metabolic syndrome, this case demonstrates the critical need for formalized perioperative protocols to optimize patient safety and outcomes.
